# Dr. George Bertram Fifield Monk

**Published:** 1915-02

**Authors:** 


DR. GEORGE BERTRAM FIFIELD MONK.—Dr. Monk was the son of Dr. Charles J. Monk, a distinguished member of the American Dental Society of Europe, who practiced in Wiesbaden, Germany, till the outbreak of the war, when he went back to his native home in England. Dr. Bertram Monk was graduated from the Dental Department of the University of Michigan in 1913, and immediately left for London, where he entered Guy's Hospital Medical School for the purpose of completing his medical course. While there he joined the Militia Company and was among the first to volunteer in the present war. Five weeks before his death he was promoted to be a Lieutenant in the Royal Warwickshire Regiment, and was killed in action December 18, 1914.

This may be chronicled as only an incident of the great war, and yet the death of this young man is peculiarly sad in view of his great promise, and the large number of friends he had made for himself in America and Europe. He was a diligent student in college and made a splendid record. His death is not only a distinct loss to dentistry, but a severe bereavement to his father on whom the exigencies of the present war have fallen with unusual hardship. We extend to the family of this brave boy our sincerest sympathy.

